# The complete coding sequence of Influenza A/Unknown/Chelyabinsk/206/H7N4

**DOI:** 10.1128/mra.00312-24

**Published:** 2024-05-20

**Authors:** S. Feoktistova, E. Degtyarev, I. Abramov, V. Artemyev, Alena V. Osipova, Irina V. Baratova, A. Kholodkova, N. Rudev, P. Volchkov, A. Deviatkin

**Affiliations:** 1 Research Institute of Autoimmune and Orphan Diseases, Federal Research Center for Innovator and Emerging Biomedical and Pharmaceutical Technologies, Moscow, Russia; 2 Сenter for personalized medicine, The MCSC named after A.S.Loginov, Moscow, Russia; 3 Department of Chemical Carcinogenesis, N.N. Blokhin NMRCO, Moscow, Russia; DOE Joint Genome Institute, Berkeley, California, USA

**Keywords:** influenza A, H7N4

## Abstract

An influenza virus strain was obtained during a bird surveillance study in 2023 near Lake Chebarkul in the Chelyabinsk region, Russia. This complete coding genome sequence of the virus sampled from the Ural region significantly expands the knowledge about the spread of the H7N4 subtype of the influenza A virus.

## ANNOUNCEMENT

Influenza A viruses (IAVs) are pathogens found in natural reservoirs such as waterfowl, bats, seals, horses, and humans (1, 2). IAVs are representatives of the genus *Alphainfluenzavirus*, family *Orthomyxoviridae*. Previous cases of both highly pathogenic and low pathogenic H7 influenza viruses in humans (3) emphasize the importance of research and sequencing to study the genetic diversity and transmission routes of viruses with high pandemic potential.

Bird fecal sample was collected near Lake Chebarkul, Chelyabinsk region, Russia during a bird surveillance study on 9 November 2023 in a tube with RNA stabilizer St-100 (Biolabmix, Russia). RNA was extracted from 100 µL of sample with an RNA column isolation kit (modified) RUplus-250 (Biolabmix, Russia) according to the manufacturer’s instructions. cDNA was obtained by SuperScript IV Reverse Transcriptase (Invitrogen, USA) using primers MBTuni-12R and MBTuni-13 (4). Further amplification of DNA was performed using PrimeSTAR GXL DNA Polymerase (Takara Bio Inc, Japan) with the same primers.

Library preparation was performed using the Raissol SG GM Plus kit from (Sesana, Russia), following the standard protocol for amplicons. We used a dual-barcode kit for Illumina (Sesana, Russia) for libraries’s indexing. Sequencing was performed on the MiSeq platform from Illumina (USA) using the MiSeq Reagent Kit v2 with 300 cycles (Illumina, USA). In total, 1,077,430 reads were obtained.

Residual adapters and bases with low-quality scores were removed using Trimmomatic version 0.39.0 (5), which removed bases from each read with a quality score of less than 20. A portion of trimmed reads (*n* = 25,000) was compared to a representative database of IAVs downloaded from the National Center for Biotechnology Information (NCBI) nucleotide database using the BLASTN algorithm to find the most similar reference sequence for each segment (*n* = 8). The Bowtie 2 tool (6) (v2.2.5) was used to align the sequencing reads to the eight references. Alignments were processed using SAMtools (7). Consensus sequences were generated for each segment using iVar (version 1.4.2) (7). The coding-complete sequences obtained had an average coverage of 1,323×. The GC content was 44.94%. At the same time, despite the high average coverage, the first 1,263 nucleotides of the PB2 segment sequence (PP506625) were not covered by sequencing reads. Therefore, this part of the PB2 segment was identified by capillary sequencing. Amplification of the incomplete coding sequence of the PB2 segment was performed using PrimeSTAR GXL DNA Polymerase (Takara Bio, Japan) with the primers PB2_1F and PB2_2R (Table 1) and further sequence reaction with all primers (Table 1). Sequencing was performed in 3,500 Genetic Analyzer (Applied Biosystems, USA). Sequencing reads of about 700 bp with overlaps of at least 100 bp were obtained (Q ≥ 30). The sequenced results were analyzed by the Ugene software (8).

**TABLE 1:** Primers for sequencing incomplete coding sequence of the PB2 segment^*a*^

Primers	Oligonucleotide sequence (5′−3′)	Length of the oligonucleotide, nucleotides
PB2_1F	AGGTCAATTATATTCAATATGGAGAG	26
PB2_2F	CAAGACGTCATCATGGAGGTCG	22
PB2_1R	CCTCGATATATACGCTGCTTG	21
PB2_2R	GCCTCAGGAGCTGATGCATAG	21
Segment name	The most similar reference sequence (the length of the sequence obtained from the newly sequenced reads, nucleotides)	The pairwise identity of the closest GenBank hit for each of the segments of A/Unknown/Chelyabinsk/206/H7N4
PB2	MF145805, A/barnacle-goose/Netherlands/2/2014 (1,050)	98.40%, A/mallard duck/Egypt/MB-D-2248C/2019(H10N4)
PB1	CY060364, A/mallard/Sweden/6/2002 (2,286)	98.02%, A/duck/Bangladesh/44407/2020(H6N1)
PA	MT819086, A/American Wigeon/Oregon/AH0038893I.3.B/2015 (2,204)	98.37%, A/Anas platyrhynchos/Belgium/11958/2018(H1N1)
HA	AF202241, A/conure/England/1234/94 (1,726)	96.79%, A/duck/Mongolia/6/2019(H7N7)
NP	CY041269, A/pink-footed goose/Netherlands/1/2006 (1,538)	98.31%, A/mallard duck/Netherlands/2/2013(H3N6)
NA	MN049528, A/mallard/Shanghai/JDS120662/2018 (1,409)	98.01%, A/mallard/Shanghai/JDS120662/2018(H10N4)
M	KF493462, A/hawk/Italy/2985/2000 (1,002)	99.40%, A/pintail/Egypt/MB-D-384C/2015(H3N6)
NS	KX978242, A/mallard duck/Netherlands/33/2014 (888)	98.76%, A/mallard duck/Netherlands/41/2015(H5N1)
Segment name	The most similar reference sequence (the length of the sequence obtained from the newly sequenced reads, nucleotides)	The pairwise identity of the closest GenBank hit for each of the segments of A/Unknown/Chelyabinsk/206 /H7N4
PB2	MF145805, A/barnacle-goose/Netherlands/2/2014 (1,050)	98.40%, A/mallard duck/Egypt/MB-D-2248C/2019(H10N4)
PB1	CY060364, A/mallard/Sweden/6/2002 (2,286)	98.02%, A/duck/Bangladesh/44407/2020(H6N1)
PA	MT819086, A/American Wigeon/Oregon/AH0038893I.3.B/2015 (2,204)	98.37%, A/Anas platyrhynchos/Belgium/11958/2018(H1N1)

^*a*^ The most similar reference sequence and the length of the sequence were obtained from the newly sequenced reads. The pairwise identity of the closest GenBank hit for each of the segments of A/Unknown/Chelyabinsk/206/H7N4.

**Fig 1:**
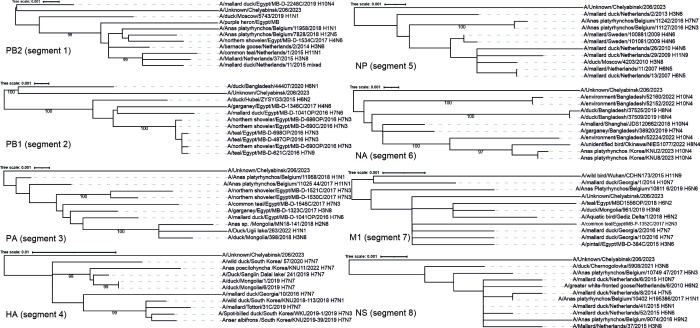
Maximum likelihood trees for eight segments of A/Unknown/Chelyabinsk/206/H7N4. Phylogenetic trees were rooted using the mid-point rooting algorithm. BLASTN algorithm was used to find 10 complete coding sequences with the highest number of identical nucleotides in comparison to each of the eight segments of A/Unknown/Chelyabinsk/206/H7N4. Maximum likelihood phylogenetic inference was inferred from the MAFFT alignment (9) using IQ-TREE (10). The best evolutionary model was automatically selected using ModelFinder (11). The phylogenetic trees were visualized using the Interactive Tree Of Life (12). Branches supported by UFBoot values above 95% are labeled with bootstrap values. Scale bars and branch lengths are the expected number of substitutions per site.

The topological structure of the phylogenetic trees (Fig. 1) showed that A/Unknown/Chelyabinsk/206/H7N4 forms a separate branch that differs from the other known strains. To date, H7N4 has been found in the USA, Canada, the Netherlands, South Korea, China, Bangladesh, Cambodia, Thailand, and Australia. At the same time, before this report, there was no information in public databases about viruses of this subtype detected in Russia.

## Data Availability

The complete coding genome sequences of A/Unknown/Chelyabinsk/206/H7N4 is available at GenBank under the accession numbers PP506625, PP506626, PP506627, PP506628, PP506629, PP506630, PP506631, and PP506632. Raw sequence data were deposited in the SRA under BioProject number PRJNA1091871 and BioSample number SAMN40615115. High throughput sequencing data are available under SRR28456454 SRA number. Capillary sequencing data are available under SRR28730451 SRA number.
